# Supplementary material for: Design of a Cereblon construct for crystallographic and biophysical studies of protein degraders
Source: Nat Commun. 2024 Oct 15;15:8885. doi: 10.1038/s41467-024-52871-9 (PMC11480361; doi:10.1038/s41467-024-52871-9)
Supplement: Supplementary file 1 — Supplementary Information [file 41467_2024_52871_MOESM1_ESM.pdf]

Supplementary Information for

Design of a Cereblon construct for crystallographic and biophysical studies of protein degraders

Alena Kroupova, Valentina A. Spiteri, Zoe J. Rutter, Hirotake Furihata, Darren, Sarath Ramachandran, Sohini Chakraborti, Kevin Haubrich, Julie Pethe, Denzel Gonzales, Andre J. Wijaya, Maria Rodriguez-Rios, Manon Sturbaut, Dylan M. Lynch, William Farnaby, Mark Nakasone, David Zollman* and Alessio Ciulli*

Corresponding authors:
David Zollman d.l.zollman@dundee.ac.uk, Alessio Ciulli a.ciulli@dundee.ac.uk

The PDF file includes:

Supplementary Note 1
Supplementary Figures 1-6
Supplementary Tables 1-5

Other Supplementary Information for this manuscript include the following:

Supplementary Data 1
Source Data

Supplementary Note 1

Molecular dynamics simulation

Relaxation protocol (pre-production run):

The following stages were involved in the relaxation process for the NPT ensemble:

1. Simulation in the NVT ensemble with Brownian dynamics at 10K with small time steps and solute non-hydrogen atoms restrained
2. Simulation in the NVT ensemble using a Langevin thermostat with:
    - a simulation time of 12 ps
    - a temperature of 10K
    - a fast temperature relaxation constant
    - velocity resampling every 1 ps
    - non-hydrogen solute atoms restrained
3. Simulation in the NPT ensemble using a Langevin thermostat and a Langevin barostat with:
    - a simulation time of 12 ps
    - a temperature of 10K and a pressure of 1 atm
    - a fast temperature relaxation constant
    - a slow pressure relaxation constant
    - velocity resampling every 1 ps
    - non-hydrogen solute atoms restrained
4. Simulation in the NPT ensemble using a Langevin thermostat and a Langevin barostat with:
    - a simulation time of 12 ps
    - a temperature of 300K and a pressure of 1 atm
    - a fast temperature relaxation constant
    - a slow pressure relaxation constant
    - velocity resampling every 1 ps
    - non-hydrogen solute atoms restrained
5. Simulation in the NPT ensemble using a Langevin thermostat and a Langevin barostat with:
    - a simulation time of 24 ps
    - a temperature of 300K and a pressure of 1 atm
    - a fast temperature relaxation constant
    - a normal pressure relaxation constant

Results and Discussion:

The RMSF of the protein residues in $CRBN^{\Delta N}$ and $CRBN^{midi}$ as observed during the MD simulation runs indicate that the two protein constructs exhibit a similar trend in their dynamic behavior (Supplementary Fig. 3a,b). RMSF differences observed at a few localized regions of $CRBN^{midi}$ *vs.* $CRBN^{\Delta N}$ are far away from the ligand binding site and are mostly on the loops and/or solvent exposed ('remarks' column of Supplementary Fig. 3 source data). The median RMSF of the protein residues in Lon, HB and TBD domains of $CRBN^{midi}$ is lower than that in the $CRBN^{\Delta N}$ construct [Median RMSF in the Lon+HB domain: 1.24 Å ($CRBN^{\Delta N}$); 0.99 Å ($CRBN^{midi}$). Median RMSF in the TBD: 1.17 Å ($CRBN^{\Delta N}$); 0.94 Å ($CRBN^{midi}$)] (Supplementary Fig.3c,d). These

observations indicate the mutations introduced in our $CRBN^{midi}$ construct perhaps impart rigidity and hence contribute towards stabilizing the protein. The RMSD plots (Supplementary Fig. 6) also indicate the higher flexibility of the $CRBN^{\Delta N}$ construct as compared to the $CRBN^{midi}$ construct. Further, the key interactions and their stabilities over the course of the MD simulations mediated by the glutarimide ring of mezigdomide with the aromatic cage of the protein remain preserved in $CRBN^{midi}$ as seen in $CRBN^{\Delta N}$ (Supplementary Fig. 3e). Overall, these computational investigations suggest that $CRBN^{midi}$ and $CRBN^{\Delta N}$ show similar profiles for protein residue fluctuations and similar ligand-mediated interactions with the TBD.

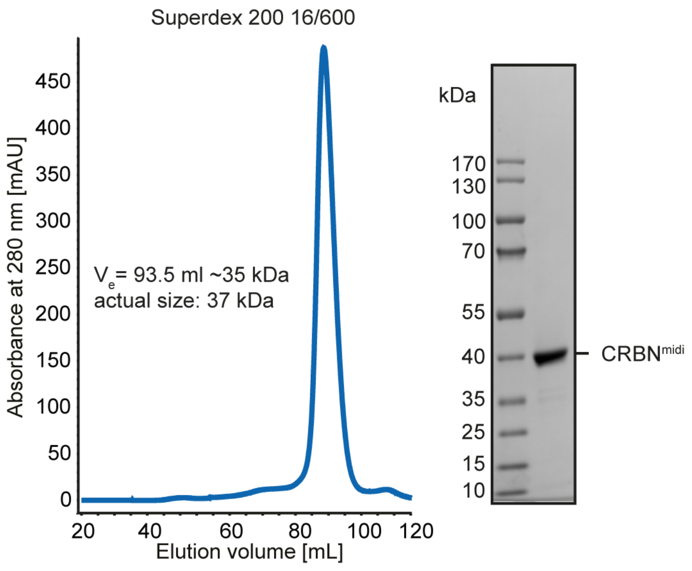

Supplementary Fig. 1: Purification of CRBN^{midi}. SEC profile of purified CRBN^{midi} (left) and SDS-PAGE analysis of the elution peak (right). The estimated size of the eluted protein calculated from a standard calibration curve is indicated. Uncropped gel is provided in the Source Data file.

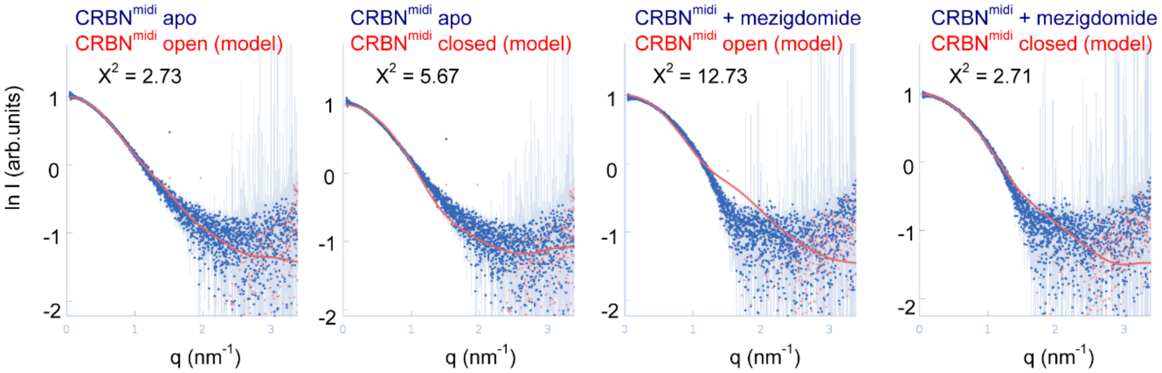

Supplementary Fig. 2: SAXS analysis of CRBN^{midi} in the absence and presence of ligands. Experimental (blue) scattering curves for apo and mezigdomide-bound CRBN^{midi}. Theoretical scattering curves (red) for open and closed CRBN^{midi} models. The scattering curve for apo CRBN^{midi} is in good agreement with the open model. In contrast, apo CRBN^{midi} does not agree as well with the closed model. CRBN^{midi}:mezigdomide is likely in a closed conformation which is demonstrated by poor agreement with the theoretical open model curve and better agreement with the theoretical closed model curve.

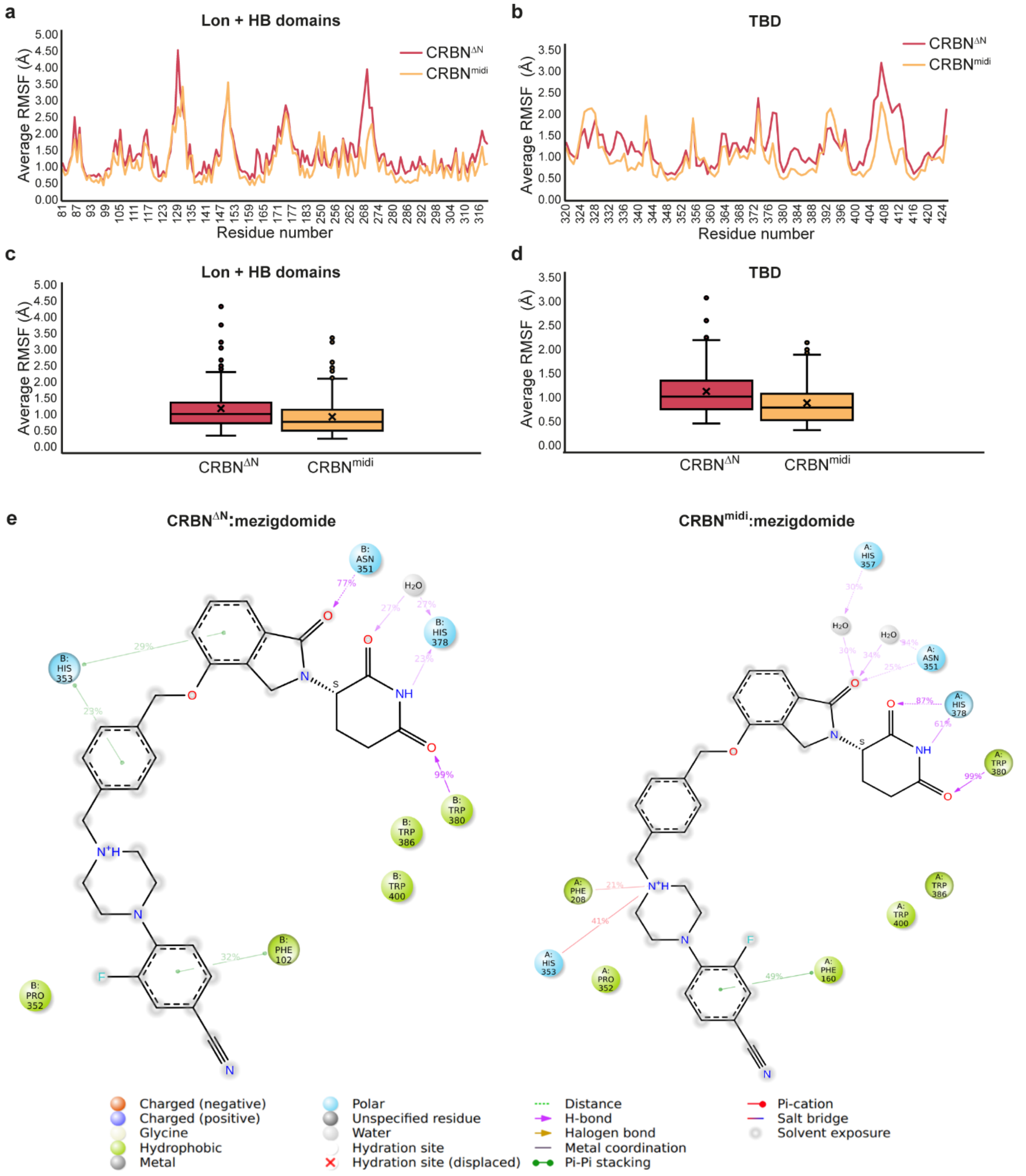

Supplementary Fig. 3: Molecular dynamics simulations. a-d) Average RMSF of the protein residues in CRBN$^{\Delta N}$ (red) *vs*. CRBN^{midi} (orange). Residue-wise distribution of average RMSF (Å) of the protein residues in the Lon+HB domains (**a**) and the TBD (**b**). Box plots showing the distribution spread of average RMSF of the protein residues in the Lon+HB domains (**c**) and the

TBD (**d**). The center line represents the median; box limits, upper and lower quartiles; whiskers, 1.5× interquartile range; circles, outliers. Source data are provided as a Source Data file. **e)** Protein-ligand interaction stability (expressed as percentage of time noted over 100 ns) as observed in a representative MD simulation run of $CRBN^{\Delta N}$:mezigdomide (Left) and $CRBN^{midi}$:mezigdomide complexes (Right).

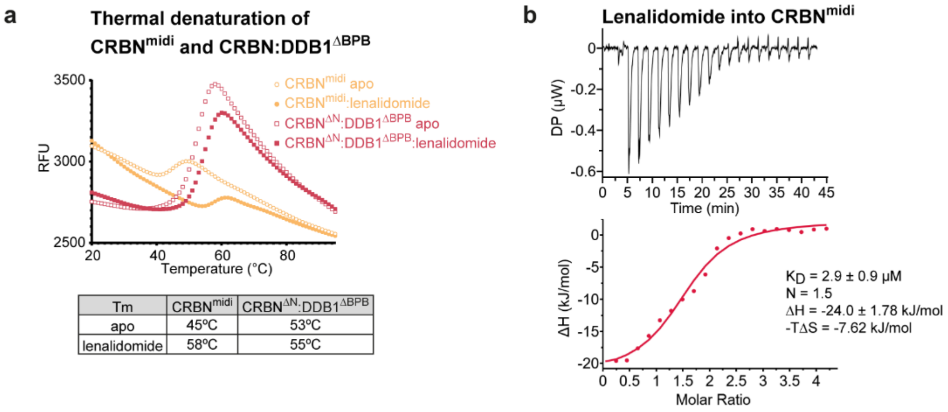

Tm	$CRBN^{midi}$	$CRBN^{\Delta N}$:$DDB1^{\Delta BPB}$
apo	45ºC	53ºC
lenalidomide	58ºC	55ºC

Supplementary Fig. 4: Biophysical analyses of lenalidomide binding to $CRBN^{midi}$. a) DSF thermal denaturation curves for $CRBN^{midi}$ (yellow) and $CRBN^{\Delta N}$:$DDB1^{\Delta BPB}$ (red) in the absence (empty circles or squares, respectively) or presence of lenalidomide (filled circles or squares). Melting temperatures (Tm) for each curve are shown in the table. **b)** ITC measurement of lenalidomide binding to $CRBN^{midi}$.

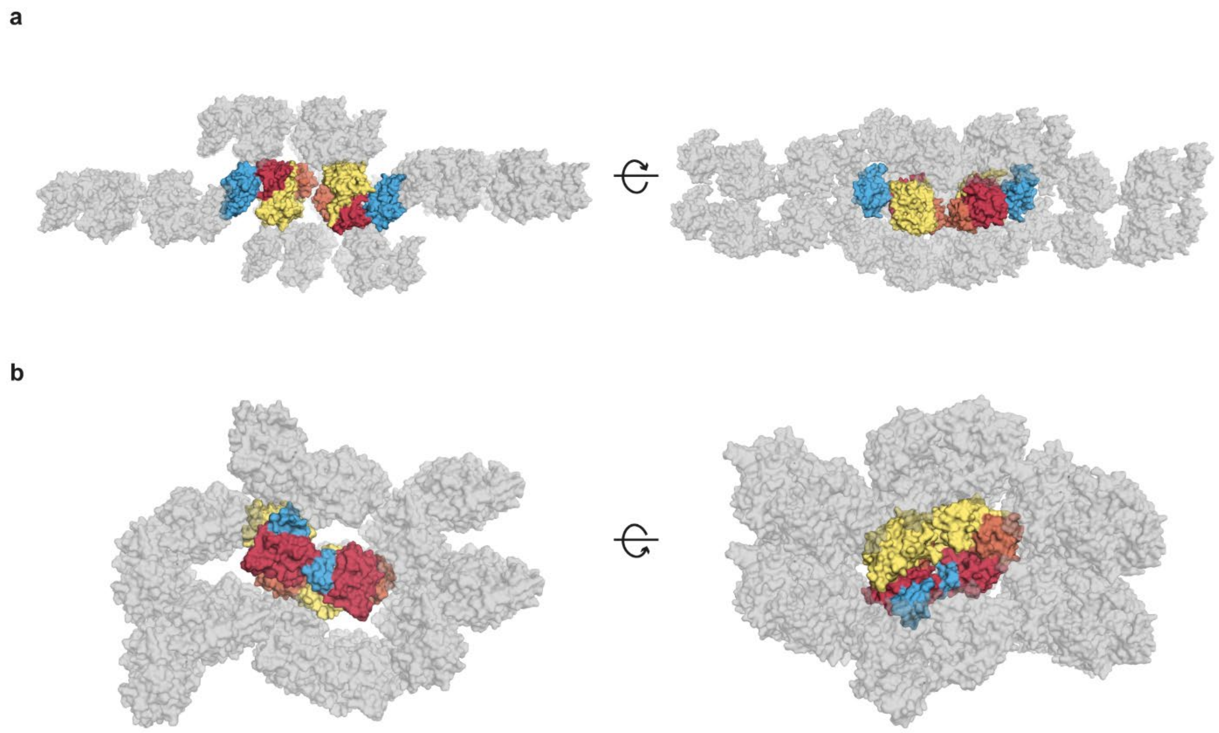

Supplementary Fig. 5: Crystal packing in ternary complex structures. a) CRBN^{midi}:CFT-1297:BRD4^{BD2} and **b)** CRBN^{midi}:mezigdomide:IKZF1^{ZF2}. The two protomers in the asymmetric unit are colored as in Fig. 3, symmetry mates are shown in grey.

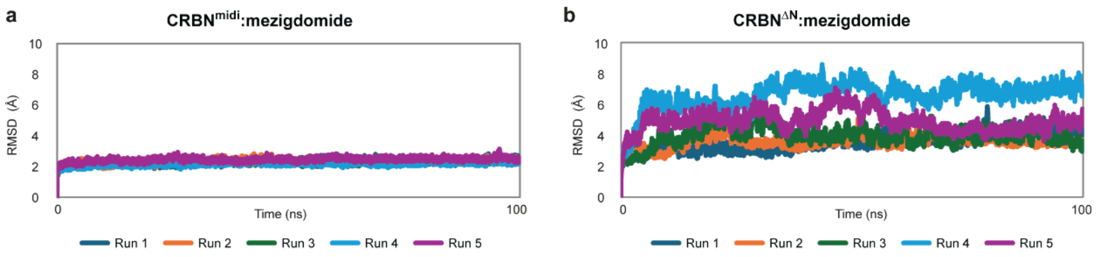

Supplementary Fig. 6. MD simulation observations. RMSD of protein heavy atoms for five different production runs over 100ns (5 x 100 ns) for the two protein constructs bound to mezigdomide. **a)** CRBN^{midi} (8R8Q, this publication) and **b)** CRBN$^{\Delta N}$ (8D7U). Source data are provided in the Source Data file.

Supplementary Table 1. Data collection and refinement statistics for CRBN^{midi} apo and ternary X-ray crystal structures.

	apo CRBN^{midi}	CRBN^{midi}:<br>mezigdomide:IKZF1^{ZF2}	CRBN^{midi}:<br>CFT-1297:BRD4^{BD2}
**Data collection**			
Space group	*C*222$_1$	*P*1211	*P*1
Cell dimensions			
a, *b*, *c* (Å)	52.03, 96.41, 148.09	53.56, 142.84, 56.69	43.88, 52.64, 130.35
α, β, γ (°)	90.0, 90.0, 90.0	90.00, 112.341, 90.00	96.44, 91.49, 99.22
Resolution (Å)	74.04 - 3.11 (3.16 - 3.11)	52.44 - 2.15 (2.23 - 2.15)	46.10 - 2.91 (2.96 - 2.91)
R_{merge}	0.410 (6.797)	0.200 (3.696)	0.333 (3.092)
$I / \sigma I$	5.0 (0.7)	6.12 (0.32)	3.3 (0.1)
$CC_{1/2}$	0.98 (0.37)	0.996 (0.343)	0.99 (0.26)
Completeness (%)	100.0 (100.0)	97.56 (80.10)	97.8 (75.6)
Redundancy	11.7 (11.6)	7.1 (7.2)	6.6 (6.0)
**Refinement**			
Resolution (Å)	74.04 - 3.11	52.44 - 2.15	43.27 - 2.91
No. reflections	6975 (688)	41733 (3417)	20285 (275)
R_{work} / R_{free}	0.267 / 0.300	0.260 / 0.287	0.248 / 0.291
No. atoms			
Protein	2119	5036	6236
Ligand/ion	1	88	122
Water	0	206	31
B-factors			
Protein	81.15	60.98	74.28
Ligand/ion	117.26	53.62	73.17
Water	-	63.50	54.08
R.m.s. deviations			
Bond lengths (Å)	0.003	0.010	0.007
Bond angles (°)	0.64	1.45	1.13
**PDB ID**	8RQ1	8RQC	8RQ9

Each dataset was collected from a single crystal. Values in parentheses are for the highest-resolution shell.

Supplementary Table 2. Data collection and refinement statistics for CRBN^{midi} binary X-ray crystal structures.

	CRBN^{midi}:mezigdomide	CRBN^{midi}:lenalidomide	CRBN^{midi}:compound **1**
**Data collection**			
Space group	$P4_32_12$	$C222_1$	$P2_12_12_1$
Cell dimensions			
a, *b*, *c* (Å)	51.05, 51.05, 267.68	51.99, 95.72, 148.25	53.51, 94.86, 147.95
α, β, γ (°)	90.0, 90.0, 90.0	90.0, 90.0, 90.0	90.0, 90.0, 90.0
Resolution (Å)	66.92 - 2.19 (2.37 - 2.19)	45.54 - 2.5 (2.54 - 2.50)	79.86 - 1.95 (1.98 - 1.95)
R_{merge}	0.244 (1.090)	0.200 (1.782)	0.144 (4.823)
$I / \sigma I$	6.4 (1.7)	5.046 (1.17)	9.7 (0.4)
$CC_{1/2}$	0.99 (0.53)	0.99 (0.42)	0.99 (0.29)
Completeness (%)	91.8 (50.9)	99.7 (96.4)	98.9 (100)
Redundancy	6.5 (4.1)	4.8 (3.7)	12.7 (10.8)
**Refinement**			
Resolution (Å)	40.59 - 2.19	45.54 - 2.5	73.98 - 1.95
No. reflections	13035 (653)	13030 (1181)	54132 (4837)
R_{work} / R_{free}	0.257 / 0.285	0.2994 / 0.342	0.252 / 0.286
No. atoms			
Protein	2405	2267	4935
Ligand/ion	43	20	42
Water	32	5	107
B-factors			
Protein	41.61	60.47	49.38
Ligand/ion	30.40	62.68	48.74
Water	41.61	39.78	41.26
R.m.s. deviations			
Bond lengths (Å)	0.009	0.015	0.003
Bond angles (°)	1.32	1.69	0.70
**PDB ID**	8RQ8	8RQA	9GAO

Each dataset was collected from a single crystal. Values in parentheses are for the highest-resolution shell.

Supplementary Table 3. Small angle X-ray scattering statistics

	**SASDU52**	**SASDU62**	**SASDU92**	**SASDVN6**	**SASDVP6**
(a) Sample Details					
**Organism**	*E. coli* BL2 (DE3)				
Source	Recombinantly expressed				
Uniprot sequence ID	*derived from human Cereblon, Uniprot Q96SW2*				
Description	$Cereblon^{midi}$, apo form	$Cereblon^{midi}$, bound to mezigdomide	$Cereblon^{midi}$, bound to lenalidomide	$Cereblon^{midi}$, bound to Boc-VcN	$Cereblon^{midi}$, bound to Boc-AcQ
Molecular mass M (kDa)	37.4	38.0	37.7	37.7	37.7
loading concentration (mg/ml)	6.5			3.4	
injection volume (µl)	50				
concentration (µM)	175			90	
Solvent composition and source	20 mM HEPES pH7.5, 500 mM NaCl, 0.5 mM TCEP				
(b) SAS data collection parameters					
Source and instrument	B21, Diamond Light Source				
Wavelength (Å)	0.9464				
Sample-detector distance (m)	3.7				
q-measurement range (Å)	4.5×10^{-3} -3.4×10^{-1}				
Radiation damage monitoring	frame-by-frame comparison				
Exposure time (s) & number	3 × 600				
Sample configuration	SEC-SAXS, Cytiva Superdex 200 Increase 3.2/300				
Sample temperature (°C)	15				
(c) Software employed for SAS data reduction, analysis and interpretation					
SAXS data processing	I(q) vs. q using Bsx cube, solvent subtraction and curve merging Chromixs				
Basic analyses: Guinier, P(r), Vp	PRIMUSqt from ATSAS 3.2.1 (Franke et al., 2017)				
Atomic structure modelling	CRYSOL 3.2.1 (Svergun et al., 1995)		-		
(d) Structural parameters					
	Guinier analysis				
I(0) (cm^{-1})	0.028 ± 3.5×10^{-5}	0.016 ± 2.1×10^{-5}	0.019 ± 2.3×10^{-5}	0.021 ± 2.1×10^{-5}	0.022 ± 2.5×10^{-5}

R_g (Å)	26.73 ± 0.06	22.79 ± 0.06	22.39 ± 0.05	23.41 ± 0.05	24.29 ± 0.05
qR_g max	1.30	1.28	1.30	1.26	1.28
Coefficient of correlation, R^2	0.82	0.76	0.81	0.84	0.62
	P(r) Analysis from AUTOGNOM				
I(0) (cm-1)	2.80 ± 2.8 × 10^{-5}	0.016 ± 1.8 × 10^{-5}	0.018 ± 1.6 × 10^{-5}	0.021 ± 1.7 × 10^{-5}	0.022 ± 2.2 × 10^{-5}
R_g (Å)	26.91 ± 0.04	22.45 ± 0.04	21.78 ± 0.02	22.93 ± 0.03	24.25 ± 0.04
d_{max} (Å)	172.46	145.02	123.16	137.14	146.92
q range (Å^{-1})	0.092 to 2.993	0.096-3.400	0.0092 to 0.3400	0.0106-0.3400	0.0097-0.3267
χ^2 (total estimate from *GNOM*)	0.83	0.79	0.85	0.80	0.82
Porod volume (Å^{-3}) (ratio V_P/calculated *M)*	60 639	56429	59090	58545	59470

Supplementary Table 4. CRBN^{midi} immobilization level to Ni-NTA chip for SPR experiments and maximal response achieved by each analyte

Sample	CRBN^{midi} immobilization level	Experimental R_{max}	Theoretical R_{max}	Experimental R_{max} / Theoretical R_{max}
**CFT1-297**	9581	196	211	93%
**BRD4^{BD2}:CFT-1297**	240	56	101	55%

Supplementary Table 5. Summary of MD simulation system setup parameters

**Protein construct**	**Ligand**	**PDB code**	**Box volume, Å³**	**Total number of atoms**	**Total number of water molecules**	**Salt concentration, NaCl (M)**
$CRBN^{midi}$	Mezigdomide	8RQ8 (this publication)	568,301	53,313	16,005	0.15
$CRBN^{\Delta N}$	Mezigdomide	8D7U	1,872,637	70,766	21,452	0.15
