# Supplementary material for: Design of a Cereblon construct for crystallographic and biophysical studies of protein degraders
Source: Nat Commun. 2024 Oct 15;15:8885. doi: 10.1038/s41467-024-52871-9 (PMC11480361; doi:10.1038/s41467-024-52871-9)
Supplement: Supplementary file 3 — Description of Additional Supplementary Files [file 41467_2024_52871_MOESM3_ESM.pdf]

Description of Additional Supplementary Files

Supplementary Data 1:

Molecular dynamics trajectories

The MD-sim_supp_data folder contains two sub-folders each containing 11 structural co-ordinate files (.pdb format), *i.e.*, total 22 pdb files in total.

10 out of the 11 structural co-ordinate files in each sub-folder are of the initial (Frame 0) and final (Frame 10000) configurations extracted from each of the 5 different runs of M.D. simulation trajectories for CRBN-midi and CRBN-d40 constructs. The other remaining files are the input structures of CRBN-midi and CRBN-d40 (PDB entry: 8D7U) that were used as input to build the systems for running M.D. simulations.

The below table provides the list of the names of the files:

S.No.	File name	Sub-folder name	Description
1	CRBNmidi_Xray_Input.pdb	CRBN-midi	The input structure to build the system for M.D. simulation of CRBNmidi:Mezigdomide complex
2	CRBNmidi_MD_run1_Frame1.pdb		Initial configuration from run1 of M.D. trajectory of CRBNmidi
3	CRBNmidi_MD_run1_Frame10000.pdb		Final configuration from run1 of M.D. trajectory of CRBNmidi
4	CRBNmidi_MD_run2_Frame1.pdb		Initial configuration from run2 of M.D. trajectory of CRBNmidi
5	CRBNmidi_MD_run2_Frame10000.pdb		Final configuration from run2 of M.D. trajectory of CRBNmidi
6	CRBNmidi_MD_run3_Frame1.pdb		Initial configuration from run3 of M.D. trajectory of CRBNmidi
7	CRBNmidi_MD_run3_Frame10000.pdb		Final configuration from run3 of M.D. trajectory of CRBNmidi
8	CRBNmidi_MD_run4_Frame1.pdb		Initial configuration from run4 of M.D. trajectory of CRBNmidi
9	CRBNmidi_MD_run4_Frame10000.pdb		Final configuration from run4 of M.D. trajectory of CRBNmidi
10	CRBNmidi_MD_run5_Frame1.pdb		Initial configuration from run5 of M.D. trajectory of CRBNmidi
11	CRBNmidi_MD_run5_Frame10000.pdb		Final configuration from run5 of M.D. trajectory of CRBNmidi

12	CRBN-d40_8D7U_Input.pdb	CRBN-d40	The input structure to build the system for M.D. simulation of CRBN-d40:Mezigdomide complex
13	CRBN-d40_8D7U_MD_run1_Frame1.pdb		Initial configuration from run1 of M.D. trajectory of CRBN-d40
14	CRBN-d40_8D7U_MD_run1_Frame10000.pdb		Final configuration from run1 of M.D. trajectory of CRBNd-40
15	CRBN-d40_8D7U_MD_run2_Frame1.pdb		Initial configuration from run2 of M.D. trajectory of CRBN-d40
16	CRBN-d40_8D7U_MD_run2_Frame10000.pdb		Final configuration from run2 of M.D. trajectory of CRBNd-40
17	CRBN-d40_8D7U_MD_run3_Frame1.pdb		Initial configuration from run3 of M.D. trajectory of CRBN-d40
18	CRBN-d40_8D7U_MD_run3_Frame10000.pdb		Final configuration from run3 of M.D. trajectory of CRBNd-40
19	CRBN-d40_8D7U_MD_run4_Frame1.pdb		Initial configuration from run4 of M.D. trajectory of CRBN-d40
20	CRBN-d40_8D7U_MD_run4_Frame10000.pdb		Final configuration from run4 of M.D. trajectory of CRBNd-40
21	CRBN-d40_8D7U_MD_run5_Frame1.pdb		Initial configuration from run5 of M.D. trajectory of CRBN-d40
22	CRBN-d40_8D7U_MD_run5_Frame10000.pdb		Final configuration from run5 of M.D. trajectory of CRBNd-40
